# Role of polyethylene glycol to alleviate lead stress in *Raphanus sativus*

**DOI:** 10.7717/peerj.18147

**Published:** 2025-01-09

**Authors:** Muhammad Sajid, Shakil Ahmed, Rehana Sardar, Aamir Ali, Nasim Ahmad Yasin

**Affiliations:** 1 Institute of Botany, University of the Punjab, Lahore, Punjab, Pakistan; 2 Department of Botany, Emerson University, Multan, Pakistan; 3 Department of Botany, University of Sargodha,, Sargodha, Punjab, Pakistan; 4 Faculty of Agricultural Sciences, University of the Punjab, Lahore, Punjab, Pakistan

**Keywords:** Antioxidant, Plant growth, Pb, PEG, Radish

## Abstract

The continuous contamination of heavy metals (HMs) in our ecosystem due to industrialization, urbanization and other anthropogenic activities has become a serious environmental constraint to successful crop production. Lead (Pb) toxicity causes ionic, oxidative and osmotic injuries which induce various morphological, physiological, metabolic and molecular abnormalities in plants. Polyethylene glycol (PEG) is widely used to elucidate drought stress induction and alleviation mechanisms in treated plants. Some recent studies have unveiled the potential of PEG in regulating plant growth and developmental procedures including seed germination, root and shoot growth and alleviating the detrimental impacts of abiotic stresses in plants. Therefore, the current study aimed to assess the effects of seed priming with various concentrations (10%, 20%, 30% and 40%) of PEG on the growth and development of radish plants growing under Pb stress (75 mg/kg soil). Lead toxicity reduced root growth (32.89%), shoot growth (32.81%), total chlorophyll (56.25%) and protein content (58.66%) in treated plants. Similarly, plants showed reduced biomass production of root (35.48%) and shoot (31.25%) under Pb stress, while 30% PEG seed priming enhanced biomass production of root (28.57%) and shoot (35.29%) under Pb contaminated regimes. On the other hand, seedlings obtained from 30% PEG priming demonstrated a notable augmentation in the concentrations of photosynthetic pigments, antioxidative activity and biomass accumulation of the plants. PEG-treated plants showed modulations in the enzymatic activities of peroxidase (PO), catalase (CAT) and superoxide dismutase (SOD). These changes collectively played a role in mitigating the adverse effects of Pb on plant physiology. Our data revealed that PEG interceded stress extenuation encompasses numerous regulatory mechanisms including scavenging of ROS through antioxidant and non-antioxidants, improved photosynthetic activity and appropriate nutrition. Hence, it becomes necessary to elucidate the beneficial role of PEG in developing approaches for improving plant growth and stress tolerance.

## Introduction

Heavy metals (HMs) are metallic elements counting lead (Pb), arsenic (As), copper (Cu), mercury (Hg), nickel (Ni), cadmium (Cd), chromium (Cr) having more than 5 gm/cm^3^ density (Koller & Saleh, 2018). Heavy metal contamination and subsequent effects of metal toxicity have become a crucial factor in deteriorating our ecosystem. Trace quantities of HMs naturally exist in the rhizospheric area. However, anthropogenic activity, rapid industrialization and unwise application of synthetic agrochemicals have enhanced HMs concentration resulting in environmental pollution (Wang et al., 2022). Metal pollution may cause negative effects on the growth and development of microbes, animals and plants.

Lead (Pb) is widely distributed in soils worldwide and is recognized as one of the world’s most hazardous metals due to its toxicity. Lead concentration in soils may range from 1–150 mg kg^−1^ (Kushwaha et al., 2018). This toxic metal severely affects cellular growth, metabolic activity and physiological progressions of plants, animals and microbes. Shi et al. (2016) revealed that Pb stress (19.11 mg kg^−1^) modulated the physiochemical activities of *Zea mays* plants. Similarly, Mani et al. (2015) found that Pb concentration in the range of 10–50 mg kg^−1^ decreased root length, shoot length, photosynthetic pigments and biomass production in chrysanthemum through enhanced biosynthesis of reactive oxygen species (ROS). Seeds sown in Pb-contaminated regimes exhibit poor germination percentage leading to poor crop stand and yield (Erdoğan & Demirhan, 2022). Moreover, Pb toxicity triggers calcium (Ca) related mechanisms thus reducing iron (Fe) interceded procedures which in turn declines seed germination, stress tolerance and biomass production (Chourasia et al., 2021). Plants affected by Pb show poor photosynthetic activity and higher dilapidation of membranous polyunsaturated fatty acyl residues leading to lipid peroxidation. Similarly, this toxic metal impedes the uptake and translocation of essential plant nutrients and water, reduces stomatal activity, declines the volume of guard cells and induces oxidative injuries through the increased synthesis of ROS. Lead decreases biosynthesis and activity of catalase. However, it may enhance the activity of peroxidase to scavenge ROS in some crop plants (Guo et al., 2020). Plants rely on their enzymatic and non-enzymatic antioxidative machinery to alleviate stress.

Seed pretreatment with some chemicals called seed priming is an economical and easy approach to improve plant growth and environmental stress tolerance (Bose et al., 2018; Kumar et al., 2021) Seedlings raised from primed seeds mitigate stress by maintaining physiochemical, metabolic and molecular activities (Taie et al., 2019; Li et al., 2021). Hydropriming is a common pre-sowing seed treatment that improves seed germination and seedlings’ vigor. Priming upregulates the expression level of stress-associated genes which empower nutrient translocation, cytoskeletal adjustment, cellular division, RNA/DNA synthesis and frequent germination of metal stress tolerant seedlings (Rajjou et al., 2012; Bewley, Bradford & Hilhorst, 2012; Manonmani, Begum & Jayanthi, 2014; Li et al., 2021). Higher activity of protein and starch hydrolyzing enzymes such as amylase and protease in the case of primed seeds assists in fast germination by improving embryo nutrition (Varier, Dadlani & Vari, 2010; Di Girolamo & Barbanti, 2012; Miransari & Smith, 2014). In the same way, priming activates the synthesis and bioactivity of antioxidative enzymes. Seed priming also augments the biosynthesis of osmolytes and membranous integrity. The higher antioxidant activity in seedlings of primed seeds mitigates stress and improves plant growth under metal-spiked circumstances (Chen et al., 2021; Kaushik et al., 2022). Furthermore, stress tolerance induction through priming agents acts as a “priming memory” which re-energizes the plant tolerance mechanism under adverse conditions (Chen & Arora, 2013; Srivastava, Suresh Kumar & Suprasanna, 2021). Besides, numerous chemical priming agents including mannitol, KNO_3_, KCI, CaCl_2_, PEG, K_3_PO_4_ and NaCI have shown enormous potential as an osmotic agent (Dadach et al., 2023). Several scientists have reported the beneficial effects of PEG as an osmopriming agent. This inert priming agent has a larger molecular size and is not toxic to the proteins and developing embryos. Stress mitigation by PEG seed priming has a trivial advantage over other stress-alleviating strategies attributable to its easy availability, ecofriendly nature and requirement in minute quantity to assuage stress tolerance and improve plant growth. It was hypothesized that priming seeds with PEG may improve plant stress tolerance (Lei et al., 2021). Radish (*Raphanus raphanistrum subsp. sativus*) is an annual or occasionally perennial dicot belonging to the family Brassicaceae. It is an economical, ancient, universal kitchen garden vegetable (Luo et al., 2020). Bioactive constituents originating from diverse plant components and extractions of radish demonstrate pharmacological attributes, showcasing efficacy in addressing a spectrum of health concerns encompassing cardiovascular disorders and microbial infections (Manivannan et al., 2019; Shirasawa et al., 2020). Radish plants exhibit vulnerability to metal-induced stress and display susceptibility to the higher concentrations of Cd and, Pb (Verma & Rani, 2021; Galhaut et al., 2014). The application of PEG assists plants in mitigating stress by regulating metabolic and physiological activities of plants (Uddin, Ullah & Nafees, 2021), improving root and shoot growth (Lei et al., 2021) and modulating the antioxidative activity of enzymes (Marimuthu, Kumutha & Sivakumar, 2018). The study regarding PEG application for plant stress tolerance is still in its early stages. Experiments for standardization of suitable PEG concentrations for stress alleviation through seed priming have not been evaluated for several crops. PEG priming under stress conditions augmented chlorophyll levels, heightened antioxidative performance, enhanced root and foliage development and intensified expression of stress-responsive genes in paddy plants (Marimuthu, Kumutha & Sivakumar, 2018). Yet, the beneficial effects of PEG seed priming on physiochemical activities and growth of Pb-stressed radish remain unknown. Consequently, it becomes mandatory to comprehend the potential of PEG in plant stress alleviation to enhance plant growth and productivity.

Therefore, the core objective of this research work was to examine the influences of Pb stress on morpho-physiological features of radish and divulge the probable mechanisms related to the PEG-interceded Pb stress alleviation in radish.

## Materials and Methods

### Seed priming and growth conditions

The seeds of radish (Variety: Mino Early syn. Diakon, White), a fast-growing Japanese cultivar commonly cultivated in many areas of Pakistan due to its non-bolting, heavy-yielding nature were obtained from RSC (Roshan Seed Corporation), Lahore, Pakistan. Firstly, the seeds were surface sterilized with 1% sodium hypochlorite for 10 min and air dried in an ambient environment after washing with distilled water thrice. Lead acetate (Pb (C_2_H_3_O_2_)_2_ as a source of Pb and polyethylene glycol (PEG) were purchased from Sigma-Aldrich (St. Louis, MO, USA). The seeds were primed in varying concentrations of PEG solutions in four beakers in which 100 ml of 10%, 20%, 30% and 40% PEG were taken and 30 seeds in each beaker were placed for 16 h, under low light exposure at 25 °C. After priming seeds were washed thrice with distilled water and dried under shade for 12 h (Shatpathy et al., 2018). Non-primed seeds were considered as control. A pot experiment was performed in a wire-house, Botanical Garden, Institute of Botany, Quid-e-Azam campus, University of the Punjab, Lahore (N 31°30′4.3236″, E 74°18′ 5.4684″). During this study, there was 18 ± 3 °C/12 ± 2 °C (day/night), 56–68% relative humidity, and 11 h photoperiod. Loam soil obtained from 0–12″land depth of the Botanical Garden, the University of Punjab, composed of 139, 510, 284 and 20 g/kg sand, clay, silt and organic matter, respectively, at 6.8 pH was used for the current study. Moisture concentration of soil at field capacity was 72% and its electrical conductivity was 0.19 dS m^−1^. While, it had 9.6 mg P, 2.39 g Si, 2.87 mg Ca, 1.65 mg K, 5.07 g N and 5.37 mg Mg in kg^−1^. Soil was contaminated with 75 mg/kg Pb and kept for 15 days before sowing of radish seeds. Afterward, 2 kg of Pb-spiked-soil and Pb-free soil were filled in 10″ plastic pots. A total of thirty pots were used with CRD. Then five primed and non-primed radish seeds were sown in the allocated pot and each treatment group was replicated three times to ensure accuracy in results. Thinning was performed after 15 days and three seedlings were allowed to grow further in each pot. Each pot was supplemented with 100 mL 50% Hoagland’s solution on each seventh day.

### Germination test

Over 10-days period, the primed seeds underwent germination defined by the emergence of a two mm long radical (Kaya et al., 2006). The germination process was meticulously monitored with observations recorded at 24-hour intervals. Each treatment underwent three replications to ensure precision in statistical analysis. The 20-day-old seedlings were systematically examined to assess various growth and germination characteristics, including overall germination percentage and seedling vigor index. Furthermore, an extensive range of parameters about growth, nutritional content and physiological traits underwent comprehensive analysis, conducted 60 days following the germination initiation. This approach was employed to ensure a thorough evaluation of the seed’s developmental progression.

The germination assessment procedures outlined in the authoritative manual of the Seed Analysis Association (Scott, Jones & Williams, 1984) were implemented to ascertain the number of viable seeds that successfully underwent germination. The quantification of germinated seeds was executed on the 9th day following seed deployment and the germination percentage was determined using the established conventional methodology (Shakirova et al., 2003) as follows:

\begin{eqnarray*}\text{Germination Percentage (GP)}= \frac{N1}{N2} \times 100 \end{eqnarray*}

Here, N1 is the number of germinated seeds, while N2 is the total number of seeds sown.

### Seedling vigor index (SV1)

The computation of the SVI was executed by employing the formula specified by the Association of Official Seed Analysis (1990) as delineated below:

\begin{eqnarray*}\text{SVI}= \left( \text{Root length}+\text{Shoot length} \right) \times GP. \end{eqnarray*}

### Assessment of morphological parameters

Following 60 days of germination, morphological parameters including plant shoot length and plant root length from three randomly selected plants of each treatment were evaluated, while pod and seed counts were done after 90 days of germination.

### Determination of fresh and dry weight of plants

The fresh shoot weight of three randomly selected seedlings (60 days following germination) from each treatment was analyzed by electric weighing balance. Before the weighing process, any surplus moisture present on the surfaces of the plants was eliminated using tissue paper. The mean values were presented as the total weight of plants in their fresh state. Subsequently, the plants were subjected to a controlled drying process within a thermal chamber set at 60 °C for 48 h. Once the drying process reaches a point where there is no discernible alteration in the weight of the plants, the dry weight is duly documented and recorded. Subsequently, the average value for all plant specimens was then computed.

### Estimation of gas exchange parameters

IRGA (Li-COR Inc, Lincoln, NE, Biosci., USA) was used to examine gas exchange properties such as net photosynthesis rate transpiration rate and stomatal conductivity, from fully stretched leaves at 10 a.m.

### Determination of chlorophyll and carotenoid pigments

Using a spectrophotometer set to measure at 440, 645 and 663 nm, the amounts of chlorophyll *a*, chlorophyll *b* and total chlorophyll were determined according to Khaleghi et al. (2012). Fresh plant leaves were crushed and mixed with 80% acetone. The test tube extract was then transferred to the cuvette. Carotenoid content was estimated by using the Lichtenthaler & Wellburn (1983) approach as follows:

\begin{eqnarray*}{C}_{\mathrm{x+c}}=(1000{A}_{470}-2.27{C}_{a}-81.4{C}_{b})/227 \end{eqnarray*}

where: C_*a*_ = chlorophyll *a* content, C_*b*_ = chlorophyll *b* content and C_x+c_ = carotenoids content

### Evaluation of plant mineral status

The plant samples (400 mg) were subjected to digestion using aqua regia at a 3: 1: 1 ratio (HNO_3_: HCLO_4_: H_2_SO_4_, V/V). After cooling, the digested components were filtered through filter paper and a final volume of 100 mL was achieved by adding distilled water. The quantification of mineral content in plant samples was determined using atomic absorption spectroscopy (nitrogen) and a flame photometer (phosphorus, potassium).

### Estimation of proline

The extraction and evaluation of free proline in radish plants were conducted following the acid ninhydrin method described by Bates, Waldren & Teare (1973). For this purpose, 100 mg frozen leaves were homogenized with two mL of sulphosalicylic acid. The samples were agitated at a rotational speed of 750 revolutions per minute (rpm) for 30 min. A 0.8 mL volume of supernatant was mixed with an equal volume of acid-ninhydrin reagent. The resultant mixture underwent vigorous agitation and was subsequently allowed to equilibrate at ambient temperature for 25 min, during which phase separation occurred. The absorbance of toluene was measured at a wavelength of 520 nm with pure toluene serving as the reference.

### Assessment of accumulation coefficient and metal tolerance index

Aqua regia an acidic mixture was used to decompose the desiccated plant material. The resulting mixture was filtered and diluted with distilled H_2_O to reach a volume of 100 mL. The concentration of Pb in both root and shoot tissues was quantified using an atomic absorption spectrophotometer. The accumulation coefficient was calculated using the following formula proposed by Al-Farraj et al. (2010):

\begin{eqnarray*}Accumulation~coefficient= \frac{Concenteration(Shootor~Root)}{Concenteration~of~soil} . \end{eqnarray*}

To calculate the metal tolerance index (MTI), the following equation was used:

\begin{eqnarray*}\%\text{MTI}= \frac{\text{Dry weight of treated plants}}{\text{Dry weight of untreated plants}} \times 100. \end{eqnarray*}

### Estimation of total soluble proteins and antioxidant enzymes

The present study employed cryogenic grinding with liquid nitrogen to process fresh plant material, thereby enabling subsequent analysis of protein content and enzymatic activities. After cryogenic grinding, the resulting finely ground plant material was immersed into a three mL phosphate buffer solution. Subsequently, the sample underwent centrifugation at 14,000 rpm for 30 min at 4 °C. The resultant supernatant from the centrifuged mixture was meticulously collected and subsequently stored at 0 °C. This strategic storage condition was implemented to facilitate the subsequent quantitative assessment of protein content, as well as enzymatic activities of POD, CAT and SOD.

The Biuret technique developed by Racusen & Johnstone (1961) was employed to estimate soluble protein concentration. To initiate the reaction, two mL of Biuret reagent was mixed with two mL of the supernatant. Subsequently, the optical density at 545 nm was measured using a spectrophotometer. The quantification of protein concentration was achieved by referencing a standard curve consisting of pre-established protein values as follows:

\begin{eqnarray*}\text{Protein contents} \left( \frac{mg}{g} \right) = \frac{\text{Curve value}\times \text{Total extract}}{\text{Extract used}\times \text{Weight of tissue used}} \times 1,000. \end{eqnarray*}

The approach established by Luck (1974) was used to evaluate POD activity. The experimental setup involved a buffer solution, guaiacol, enzyme extract and hydrogen peroxide solution. The quantification of POD was achieved by ascertaining the duration necessary to attain a 0.1-unit increment in absorbance at 240 nm.

\begin{eqnarray*}\text{Peroxid aseactivity} \left( \frac{mg}{g} \right) = \frac{\text{Absorbance}\times df}{\text{Weight of tissue used}\times EU} \times 1000. \end{eqnarray*}

df = dilution factor EU = extraction unit

The CAT activity assessment was conducted using the Beers & Sizer (1952) technique, in the presence of two buffer solutions. For this purpose, a reaction mixture comprising 2.9 mL of buffer B and 0.2 mL of enzyme extract was used. In contrast, the control group contained just three mL of buffer A. The duration required for the reduction of absorbance (at a wavelength of 240 nm) from 0.45 to 0.40 was utilized for CAT activity evaluation. This activity was subsequently quantified as units per mL of the enzyme. This method ensures a rigorous and standardized approach, contributing to the robustness and reliability of the scientific investigation.

\begin{eqnarray*}\text{Catalase activity} \left( \frac{\text{units}}{\text{mLenzyme}} \right) = \frac{3.45\times df}{Min\times 0.1} . \end{eqnarray*}

Maral, Puget & Michelson (1977) conducted a study wherein they employed spectrophotometric analysis to measure the activity of SOD. This was achieved by evaluating its capacity to hinder the photochemical reduction of nitroblue tetrazolium. For this purpose, two test tubes containing a mixture of sodium cyanide, NBT methionine, riboflavin and EDTA as a substrate were employed in the technique. The absorbance of the illuminated tube at a wavelength of 560 nm was compared to that of the non-illuminated mixture. The measurement of SOD activity was conducted in mg of protein.

\begin{eqnarray*}\%\text{Inhibition}= \frac{\text{absorption of control sample}-\text{absorption of experimental sample}}{\text{absorption of experimental sample}} \times 100. \end{eqnarray*}

### Determination of hydrogen peroxide and malondialdehyde content

Hydrogen peroxide extraction from plant tissues was induced following the methodology proposed by Patterson, MacRae & Ferguson (1984). The pulverization of fresh leaves was conducted, in conjunction with the utilizing TCA (trichloroacetic acid) and activated charcoal. The sample underwent centrifugation at 3,000 rpm for 20 min at a temperature of 4 °C. The filtrate was then divided into one mL aliquots. One of these containers was filled with 81 g of CAT and kept at ambient temperature for 10 min. A one mL volume of colorimetric reagent was introduced to both the aliquots and allowed to incubate for 10 min at a temperature of 30 °C. The spectrophotometric measurement was conducted to determine the absorbance at 505 nm.

1g of foliage was subjected to vortex with the solutions of thiobarbituric acid (six mL) and trichloroacetic acid and for 15 min underwent incubation at 95 °C. Subsequently, the mixture was cooled and then centrifuged (10,000 × g) for 20 min, which finally led the supernatant to a spectrophotometer for quantification of malondialdehyde (MDA) content (Zheng et al., 2012).

### Statistical analysis

The data obtained represents the mean value of three replicates. RStudio software was employed to conduct principal component analysis (PCA) and determine Pearson correlation coefficients among the observed variables. SPSS version 20 was used to calculate the analysis of variance. Subsequently, Tukey’s Honestly Significant Difference (HSD) test was employed to assess the treatment means, enabling the ranking and comparison of significantly distinct treatments at a probability level of *P* < 0.05.

## Results

### Seed germination and seedling vigor index

An observable increase in the germination % and seedling vigor index (SVI) has been noted due to the Pb and PEG applications. The absence of the PEG application caused a significant decline in the germination % (36.6%) and the SVI (59.13%) was noted under Pb stress conditions compared to control plants. Table 1 presents the observed fluctuations in germination percentage and morphological parameters in response to the imposition of Pb-induced stress. The seedlings subjected to Pb-induced stress exhibited a reduced germination percentage (47.7%) compared to plants in the control group. Values for maximum growth performance (GP) and SVI were recorded at the PEG-30% treatment, exhibiting elevated germination compared to both the control group and Pb-contaminated treatment, as well as other PEG treatments. The germination rate in the PEG-30% treatment showed a notable increase of 19.04% compared to the control. Under the influence of Pb stress, reduction is observed in various growth attributes compared to the control group. Specifically, there is a reduction of shoot length (48.83%) and root length (32.89%) under Pb stress. The recorded data under Pb stress conditions revealed that shoot and root length significantly increased in the presence of PEG-30%. Specifically, a remarkable increase of 62.79% in shoot length and 50.98% in root length was observed compared to the conditions solely subjected to Pb stress.

**Table 1:** Effect of PEG on the growth and biomass production of *R. sativus* under lead stress.

Treatments	Growth Traits								
	**Shoot length** **(cm)**	**Root length** **(cm)**	**Shoot FW** **(g plant**^−1^)	**Root FW** **(g plant**^−1^)	**Shoot DW** **(g plant**^−1^)	**Germination percentage**	**Seedling vigor index**	**No of pods/plant**	**No of seeds/pod**
C	6.4 ± 0.43 ab	7.6 ± 0.29 ab	0.32 ± 0.02 ab	0.31 ± 0.04 a	0.05 ± 0.001 ab	70 ± 0.88 b	9.3 ± 0.32 b	9.6 ± 0.88 ab	10 ± 0.57 abc
Pb	4.3 ± 0.43 d	5.1 ± 0.26 d	0.22 ± 0.00 c	0.2 ± 0.02 a	0.02 ± 0.001 f	36 ± 1.98 e	3.8 ± 0.23 f	3.3 ± 0.33 d	3 ± 0.88 f
PEG-1	4.5 ± 0.29 cd	7.1 ± 0.14 abc	0.26 ± 0.01 bc	0.26 ± 0.01a	0.03 ± 0.001 def	59 ± 1.20 cd	6.9 ± 0.21 e	6.0 ± 0.57 cd	7 ± 0.57cde
PEG-2	5.3 ± 0.47 bcd	7.2 ± 0.23 abc	0.28 ± 0.00abc	0.28 ± 0.01 a	0.03 ± 0.001 ef	67 ± 1.35 bc	8.1 ± 0.31 cd	7.3 ± 0.33 abc	9 ± 0.57 bcd
PEG-3	6.1 ± 0.08 abc	7.4 ± 0.23 abc	0.33 ± 0.02 ab	0.27 ± 0.03 a	0.05 ± 0.001 a	85 ± 1.19 a	11.2 ± 0.13 a	10.0 ± 0.57 ab	11 ± 0.57 ab
PEG-4	4.4 ± 0.14 d	6.5 ± 0.11 c	0.29 ± 0.01 abc	0.26 ± 0.00 a	0.03 ± 0.001 cde	65 ± 2.55 bc	7.5 ± 1.17 de	6.6 ± 0.88 bcd	4 ± 1.20 ef
PEG-1 + Pb	5.4 ± 0.17 abcd	7.2 ± 0.05 abc	0.29 ± 0.00 abc	0.25 ± 0.00 a	0.04 ± 0.002 bc	57 ± 0.98 e	7.1 ± 0.14 de	5.6 ± 0.66 cd	7 ± 0.66 cde
PEG-2 + Pb	6.2 ± 0.43 ab	7.3 ± 0.31 abc	0.3 ± 0.00 ab	0.26 ± 0.00 a	0.04 ± 0.002 abc	65 ± 1.14 bc	9.0 ± 0.19 bc	7.0 ± 1.0 abc	9 ± 0.57 bcd
PEG-3 + Pb	7 ± 0.11 a	7.7 ± 0.23 a	0.34 ± 0.01a	0.28 ± 0.02 a	0.05 ± 0.00 ab	84 ± 2.21 a	11.6 ± 0.19 a	10.3 ± 0.88a	13 ± 0.57 a
PEG-4 + Pb	3.9 ± 0.29 d	6.6 ± 0.12 bc	0.3 ± 0.00 ab	0.25 ± 0.00 a	0.04 ± 0.00 cd	61 ± 1.39 cd	7.1 ± 0.19 e	7.0 ± 0.57 abc	6 ± 0.57 def

**Notes.**

The data represent the mean standard deviation (SD) of three replicates. Distinct lowercase letters indicate a notable difference between the treatments at a significance level of *P* < 0.05.

C control group Pb 75 mg/kg PEG-1 10% PEG PEG-2 20% PEG PEG-3 30% PEG PEG-4 40% PEG PEG-1-Pb PEG-10%+Pb PEG-2+Pb PEG-20%+Pb PEG-3+Pb PEG-30%+Pb PEG-4+Pb PEG-40%+Pb

### Biomass assessment

The radish plants subjected to Pb-induced stress showed a notable decline in the fresh weights of both their shoot and root, with a reduction of approximately 32.89% and 35.48%, respectively, compared to the control seedlings. The data exhibited that the PEG-primed seeds revealed a distinct positive impact on fresh and dry biomass production, in contrast to plants subjected to Pb-induced stress. Lead stress reduced shoot and root biomass production by 60% and 50%, respectively, in seedlings. Growth parameters increased with increased PEG concentrations. Nevertheless, it is important to mention that an increase in the concentration of PEG (up to 40%) resulted in a slight decrease in growth. This decline, though relatively minor, was still comparable to the control group and showed no significant difference when compared to plants subjected to Pb-stress. The application of PEG-30% significantly enhanced the fresh weight of the shoot (54.54%) and root (40%) compared to plants that did not undergo priming and were exposed to Pb-stress. The seed that underwent priming with PEG-30% showed a notable reduction in fresh and dry biomass production compared to the control group (Table 1). Maximum pods and highest seed number per pod in treated plants were counted in PEG-30%+Pb treated plants, which were 7.29% and 30% higher, respectively, compared with the plants grown in a stress-free condition (control). Pb toxicity resulted in a notable decrease in the quantity of pods and the number of seeds per pod. The observed pod count per plant in Pb stress conditions exhibited a 65.62% reduction compared to the number of pods per plant in the control group. Additionally, the number of seeds per pod in plants treated with Pb was 70% lower than that of the control group (Table 1).

### Estimation of gas exchange parameters

**Figure 1:**
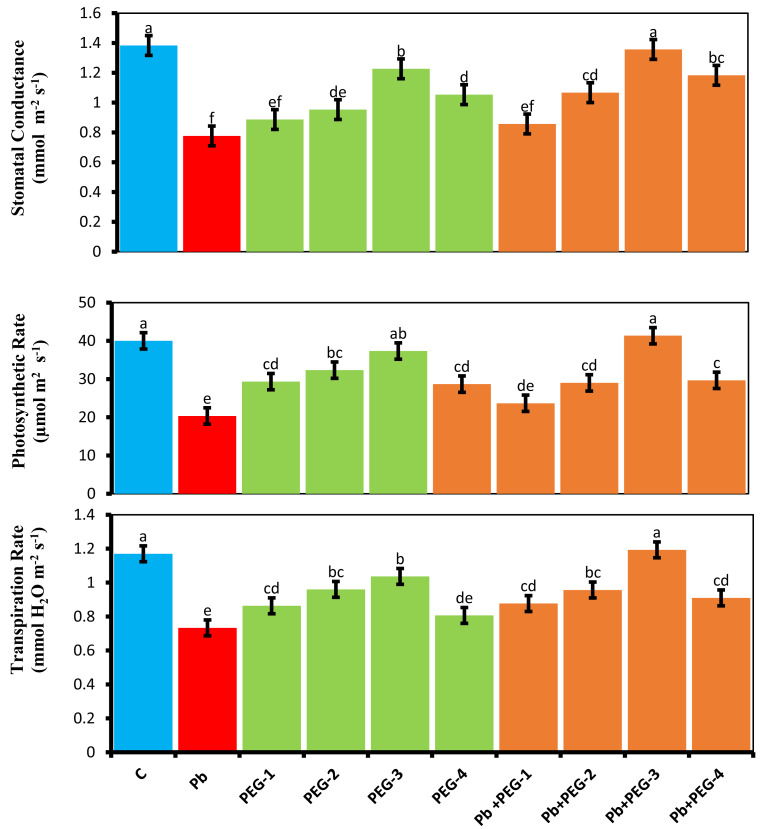
Effect of PEG on gaseous exchange parameters of *R. sativus* seedlings under lead stress. The data represent the mean standard deviation (SD) of three replicates. Distinct lowercase letters indicate a notable difference between the treatments at a significance level of *P* < 0.05. C, control group; Pb, 75 mg/kg; PEG-1, 10% PEG; PEG-2-20% PEG, PEG-3, 30% PEG; PEG-4-40% PEG, PEG-1-Pb-PEG-10%+Pb; PEG-2+Pb-PEG-20%+Pb, PEG- 3+Pb-PEG-30%+Pb; PEG-4+Pb, PEG-40%+Pb.

Plants subjected to Pb stress exhibited a notable reduction in gas exchange traits, including net photosynthetic activity (*A*), transpiration rate (*Ei*) and stomatal conductivity (*gs*), as compared to the control group. Specifically, the observed decreases were 46.15%, 36.36%and 49–25% for stomatal conductivity, transpiration rate and net photosynthetic rate, respectively, compared to the control plants. Polyethylene glycol (PEG) primed seeds exhibited enhanced gas exchange parameters, thereby mitigating the unfavorable effects of Pb in radish. Specifically, when exposed to a combination of PEG-30% and Pb, these plants demonstrated an 85.71% increase in stomatal conductivity, 57.14% enhancement in transpiration and a 93.59% increase in net photosynthetic rate in contrast, plants that were solely treated with Pb did not exhibit such improvements (Fig. 1).

### Determination of photosynthetic pigments

The radish plants exhibit an apparent decline in their photosynthetic pigments when subjected to soil contaminated with Pb. Compared to control, Pb reduced chlorophyll *b* synthesis (0.2 mg/g FW) more than chlorophyll *a* (0.4 mg/g FW). Our results showed a decrease of 45.94% in carotenoids, 56.25% in total chlorophyll and 60% in chlorophyll *b* content. Significant enrichments were noted in the levels of chlorophyll content and carotenoids within the plant that underwent pre-treatment with PEG, under both Pb-stressed and non-stressed soil conditions.

The application of PEG has been found to enhance the synthesis of photosynthetic content. In particular, the best results were obtained by seed priming with PEG at a concentration of 30%. This concentration increased Chl *a* by 60%, Chl *b* by 61%, total chlorophyll by 56.25% and carotenoids by 58.33% under Pb-induced stress (Table 2).

**Table 2:** Effect of PEG on photosynthetic pigments, total chlorophyll and carotenoids of *R. sativus* under lead stress.

Treatments	Chl *a* (mg/g FW)	Chl *b* (mg/g FW)	Total Chlorophyll (mg/g FW)	Carotenoids (mg/g FW)
C	0.9 ± 0.00 b	0.5 ± 0.01 a	1.6 ± 0.01 ab	3.7 ± 0.14 a
Pb	0.4 ± 0.03 e	0.2 ± 0.01e	0.7 ± 0.01 c	2.0 ± 0.00 f
PEG-1	0.8 ± 0.01 c	0.3 ± 0.00 cd	1.2 ± 0.00 bc	4.5 ± 0.01c
PEG-2	0.8 ± 0.01 c	0.3 ± 0.00 cd	1.3 ± 0.01abc	4.7 ± 0.02 b
PEG-3	1.0 ± 0.01 a	0.4 ± 0.01 bc	1.6 ± 0.01 ab	4.9 ± 0.01 a
PEG-4	0.6 ± 0.01 d	0.3 ± 0.01de	1.6 ± 0.01 abc	3.1 ± 0.01 a
PEG-1 + Pb	0.6 ± 0.01 d	0.3 ± 0.00de	1.1 ± 0.01 abc	3.9 ± 0.01 d
PEG-2 + Pb	0.8 ± 0.03 bc	0.3 ± 0.03de	1.3 ± 0.01 ab	4.3 ± 0.02 b
PEG-3 + Pb	1.0 ± 0.02 a	0.5 ± 0.01 ab	1.6 ± 0.01a	4.8 ± 0.01 a
PEG-4 + Pb	0.6 ± 0.02 d	0.3 ± 0.01de	1.0 ± 0.01 ab	4.6 ± 0.01 e

**Notes.**

The data represent the mean standard deviation (SD) of three replicates. Distinct lowercase letters indicate a notable difference between the treatments at a significance level of *P* < 0.05.

C control group Pb 75 mg/kg PEG-1 10% PEG PEG-2 20% PEG PEG-3 30% PEG PEG-4 40% PEG PEG-1-Pb PEG-10%+Pb PEG-2+Pb PEG-20%+Pb PEG-3+Pb PEG-30%+Pb PEG-4+Pb PEG-40%+Pb

### Evaluation of plant mineral status

Table 3 showed data on nitrogen (N), phosphorus (P) and potassium (K) uptake found in the leaf tissues of *R. sativus* plants. Plants subjected to Pb treatment exhibited a reduction in their N, P and K levels. It was noted that the N content decreased by 43.47%, the P content by 28.96%and the K content by 30.35% compared to the control plants. Leaf samples exhibited significantly elevated N, P and K levels in plants subjected to PEG-30%+Pb treatment over stress plants. PEG-30%+Pb upsurges the N by 50.93%, P by 46.4% and K by 38.18% over Pb-induced seedlings.

**Table 3:** Effect of PEG on nitrogen, phosphorus and potassium of *R. sativus* under lead stress.

Treatments	N (mg/g DW)	P^+^ (mg/g DW)	K^+^ (mg/g DW)
C	41.6 ± 0.61 b	11.7 ± 0.17 b	43.1 ± 1.21 bc
Pb	23.5 ± 1.03 e	6.7 ± 0.15 h	30.6 ± 1.02 e
PEG-1	27.8 ± 1.28 de	8.5 ± 0.17 f	35.2 ± 1.56 de
PEG-2	33.5 ± 1.07 cd	9.7 ± 0.12 e	39.9 ± 0.56 cd
PEG-3	41.1 ± 0.73 b	10.6 ± 0.08 cd	46.3 ± 2.23 ab
PEG-4	26.9 ± 1.21 e	7.6 ± 0.20 g	30.5 ± 0.95 e
PEG-1 + Pb	38.0 ± 0.94 bc	10.5 ± 0.17 de	35.0 ± 0.84 de
PEG-2 + Pb	40.4 ± 1.27 b	11.4 ± 0.17 bc	43.0 ± 0.82 bc
PEG-3 + Pb	47.9 ± 1.56 a	12.5 ± 0.18 a	49.5 ± 1.10 a
PEG-4 + Pb	33.5 ± 1.89 cd	7.5 ± 0.18 g	31.9 ± 1.44 e

**Notes.**

The data represent the mean standard deviation (SD) of three replicates. Distinct lowercase letters indicate a notable difference between the treatments at a significance level of *P* < 0.05.

C control group Pb 75 mg/kg PEG-1 10% PEG PEG-2 20% PEG PEG-3 30% PEG PEG-4 40% PEG PEG-1-Pb PEG-10%+Pb PEG-2+Pb PEG-20%+Pb PEG-3+Pb PEG-30%+Pb PEG-4+Pb PEG-40%+Pb

### Assessment of Pb uptake

Table 4 indicated that plants grown in Pb-enriched soil showed enhanced Pb uptake by 3.9 µg/g DW in plant roots and 0.12 µg/g DW in plant shoots. Nevertheless, PEG treatment inhibited Pb translocation and uptake in both roots and shoots. Pb concentrations in roots grown under 10, 20, 30and 40% PEG were 3.48, 2.40, 3.77 and 2.73 µg/g DW and in PEG-treated plant shoots, they were 0.336, 0.493, 0.246 and 0.166 µg/g DW. PEG 20 and 30% reduced the Pb uptake by 50% and 21% in roots and 16.86% and 53.51% in shoots, respectively.

**Table 4:** Effect of PEG seed priming on Pb uptake, accumulation coefficients and metal tolerance index, vigor index, number of Pods per plant, and number of seeds pod^−1^ of *R. sativus* under lead stress.

Treatments	Pb uptake in root (µg/g 1 DW)	Pb uptake in shoot (µg/g 1 DW)	AC	MTI
C	0	0	0	0
Pb	3.9 ± 0.67 a	0.59 ± 0.10 a	0.05 ± 0.008 a	42.3 ± 0.02 d
PEG 10%	0	0	0	0
PEG 20%	0	0	0	0
PEG 30%	0	0	0	0
PEG 40%	0	0	0	0
Pb+PEG 10%	3.5 ± 0.35 ab	0.33 ± 0.06 ab	0.05 ± 0.004 ab	67.6 ± 3.75 c
Pb+PEG 20%	2.4 ± 0.25 b	0.49 ± 0.11 ab	0.03 ± 0.003 b	125.6 ± 4.40 a
Pb+PEG 30%	3.8 ± 0.22 ab	0.24 ± 0.03 ab	0.05 ± 0.002 ab	102.6 ± 3.17 b
Pb+PEG 40%	2.7 ± 0.15 b	0.16 ± 0.03 b	0.04 ± 0.002 b	65 ± 2.88 c

**Notes.**

The data represent the mean standard deviation (SD) of three replicates. Distinct lowercase letters indicate a notable difference between the treatments at a significance level of *P* < 0.05.

C control group Pb 75 mg/kg PEG-1 10% PEG PEG-2 20% PEG PEG-3 30% PEG PEG-4 40% PEG PEG-1-Pb PEG-10%+Pb PEG-2+Pb PEG-20%+Pb PEG-3+Pb PEG-30%+Pb PEG-4+Pb PEG-40%+Pb

### Assessment of metal tolerance index and accumulation coefficient

The plants cultivated in soil contaminated with Pb exhibited AC of 0.05 and MTI of 42.33%. Application of 20% PEG and 30% PEG resulted in significantly increased MTI values by 66.32% and 58.77%, respectively compared to plants affected by Pb contamination. The seed primed with 20% PEG under Pb stress, resulted in a significant reduction of the apparent conductivity (AC) value by 40% (Table 4).

### Estimation of antioxidant enzyme activities

**Figure 2:**
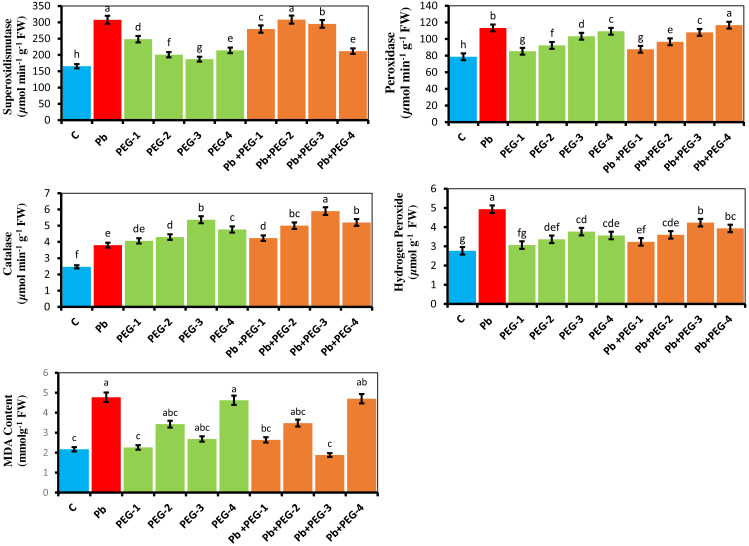
Effect of PEG on SOD, POD, CAT, H_2_O_2_ and MDA activity of *R. sativus* seedlings under lead stress. The data represent the mean standard deviation (SD) of three replicates. Distinct lowercase letters indicate a notable difference between the treatments at a significance level of *P* < 0.05. C, control group; Pb, 75 mg/kg; PEG-1, 10% PEG; PEG-2-20% PEG; PEG-3, 30% PEG; PEG-4-40% PEG; PEG-1-Pb-PEG-10%+Pb, PEG-2+Pb-PEG-20%+Pb, PEG- 3+Pb-PEG-30%+Pb, PEG-4+Pb, PEG-40%+Pb.

The current investigation explains the physiological reactions of antioxidant enzymes in the presence of Pb and PEG treatments. According to studies, the utilization of various concentrations of PEG (10%, 20%, 30% and 40%) in isolation, as well as in combination with Pb, exhibited a notable enhancement in the superoxide dismutase (SOD) activity of the radish plant when compared to the control group. Similarly, the levels of catalase (CAT), hydrogen peroxide (H_2_O_2_), malondialdehyde (MDA) and peroxidase (POD) activities were found to be elevated. The comparative investigation among plants treated with Pb and control plants demonstrated that the Pb significantly upsurge the levels of SOD, POD, CAT, H_2_O_2_ and MDA by 86.08%, 44.07%, 54.47%, 81.48% and 54.71% respectively (Fig. 2). The Pb+ PEG-20% treatment revealed the most elevated SOD activity. This treatment demonstrated a notable increase of 86.50% compared to the control group and a slight increment of 0.22% compared to plants treated solely with Pb. The Pb+PEG-40% treatment exhibited the highest level of POD activity, surpassing that of plants treated with Pb by 2.91% and the control by 48.34%. The plants subjected to the combination of Pb+PEG-30% showed the most significant elevation in CAT activity (55.26%), over the plants exposed solely to Pb stress. Conversely, the plants treated exclusively with Pb demonstrated the highest levels of H_2_O_2_ and MDA contents showing a considerable 81.48% and 54.71% increase respectively compared to the control treatment.

### Estimation of proline content

**Figure 3:**
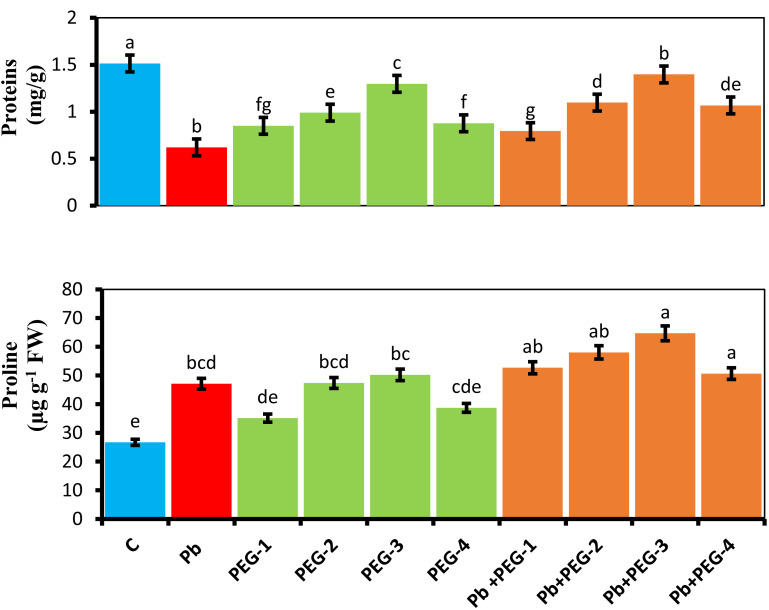
Effect of PEG on total soluble proteins, proline contents of *R. sativus* seedlings under lead stress. The data represent the mean standard deviation (SD) of three replicates. Distinct lowercase letters indicate a notable difference between the treatments at a significance level of *P* < 0.05. C, control group; Pb, 75 mg/kg; PEG-1, 10% PEG; PEG-2-20% PEG; PEG-3, 30% PEG; PEG-4-40% PEG, PEG-1-Pb-PEG-10%+Pb; PEG-2+Pb-PEG-20%+Pb, PEG- 3+Pb-PEG-30%+Pb; PEG-4+Pb, PEG-40%+Pb.

When PEG was applied as a seed pre-treatment, proline concentration in radish plants under Pb stress conditions significantly increased. Under Pb-stress, PEG-30% showed the highest proline concentration, measuring about 5.24 mg/g. The findings of this experiment indicate that the proline concentration of radish plants was significantly higher in the treatment group when different PEG concentrations (10%, 20%, 30% and 40%) were used alone or in combination with Pb. Upon subjecting plants solely to Pb, a substantial and prominent increase in proline content of 43.28% was observed compared to the control group (Fig. 3).

### Estimation of soluble protein content

The presence of Pb hinders the plant’s ability to synthesize proteins, leading to a subsequent decrease in various growth parameters. Upon comparing plants subjected solely to Pb treatment with the control group, a notable and significant reduction of 60% in their protein content was observed. Application of pre-treatment of PEG exhibited a distinct positive impact on the protein concentrations in radish plants subjected to Pb stress conditions. PEG-30% demonstrates the highest protein concentration by 1.3 mg/g under Pb-induced stress. This concentration closely resembles the control values, indicating the Pb mitigating effects of PEG (Fig. 3).

### Correlation between various growth and physio-biochemical attributes with Pb uptake and accumulation

**Figure 4:**
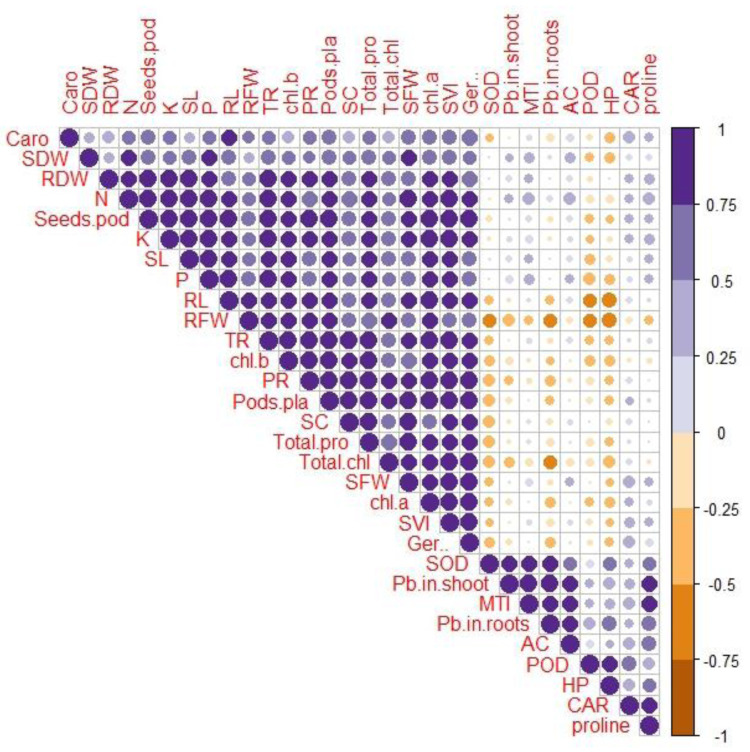
Correlation between growth and physio-biochemical attributes of PEG primed *R. sativus* grown in lead contaminated soil. The figure employs various abbreviations to represent specific parameters, includes: SL (shoot length), RL (root length), SFW (shoot fresh weight),RFW (root fresh weight), SDW (shoot dry weight), RDW (root dry weight), SC (stomatal conductance), PR (photosynthetic rate), TR (transpiration rate), T proteins (soluble protein content), SOD (Superoxide dismutase Activity), POD (peroxidase activity), Caro (carotenoids), CAT (catalases activity), HP (hydrogen peroxides activity), Caro (carotenoids), chl b (chlorophyll b), chl *a* (chlorophyll a), TC (total chlorophyll), pods/pla (pods per plants), seeds/pod (seeds per plants), SVI (seed vigor index), N (nitrogen), P (phosphorus), K (potassium), Pro (proline content in radish plants), Pb in root (lead in radish Roots), Pb in shoot (lead in radish shoot), AC (accumulation coefficient), MTI (metal tolerance index), GP (germination percentage).

A Pearson’s correlation analysis was employed to identify the association between the growth and physio-biochemical characteristics of PEG-treated *R. sativus* cultivated in soil contaminated with Pb (Fig. 4). The concentration of Pb in the shoot exhibited a positive correlation with the accumulation coefficient in the shoot and the proline content in plants of the species *R. sativus*. In contrast, the concentration of Pb in the shoot exhibited a negative correlation with various factors including metal tolerance index, potassium, root dry weight, total phenol content, stomatal conductance, germination percentage, total chlorophyll, transpiration rate, shoot dry weight, photosynthetic rate, protein content, fresh weight of root and shoot, root length and carotenoids. According to these findings, PEG showed efficacy in reducing the negative impacts of Pb stress by enhancing the absorption of vital nutrients and modifying antioxidant enzymes.

### Principal component analysis

**Figure 5:**
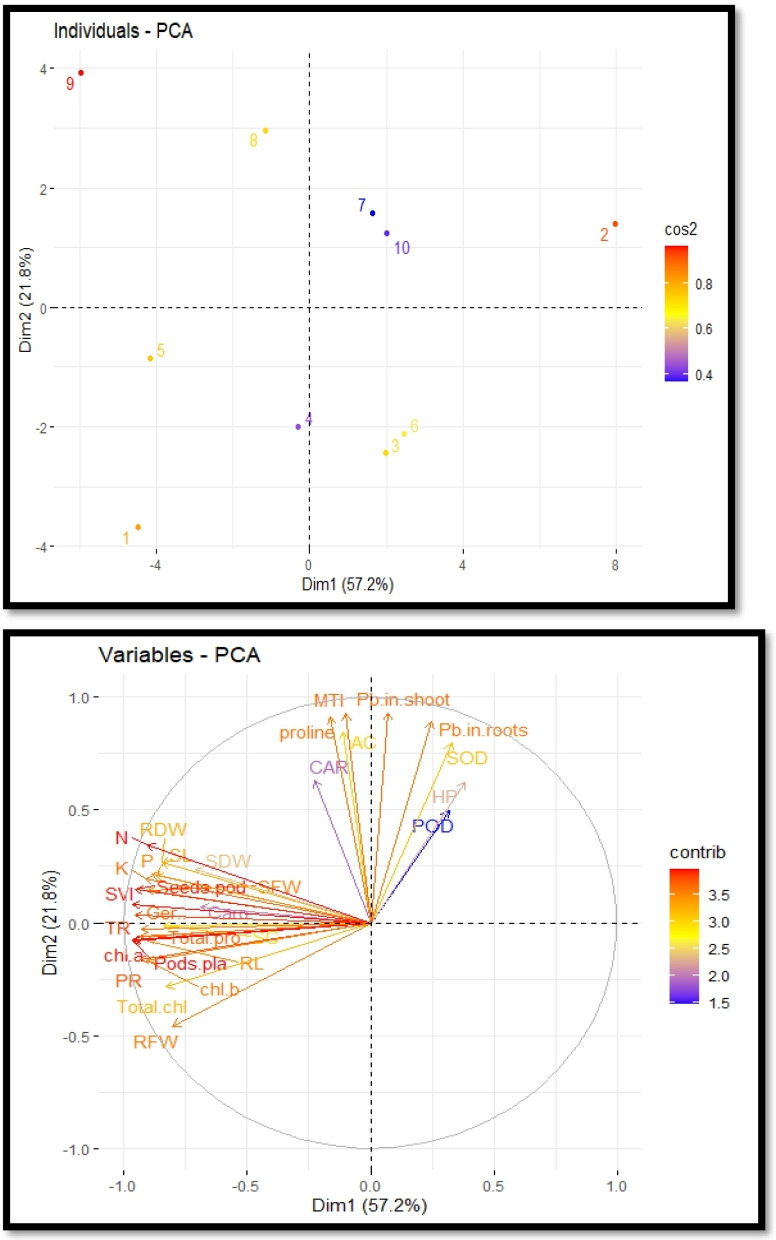
Principal component analysis plots to illustrate the relationship between growth and physio-biochemical characteristics of PEG treated *R. sativus* cultivated in lead-contaminated soil. The figure employs various abbreviations to represent specific parameters, include: SL (shoot length), RL (root length), SFW (shoot fresh weight), RFW (root fresh weight), SDW (shoot dry weight), RDW (root dry weight), SC (stomatal conductance), PR (photosynthetic rate), TR (transpiration rate), T proteins (soluble protein content), SOD (superoxide dismutase Activity), POD (peroxidase activity), Caro (carotenoids), CAT (catalases activity), HP (Hydrogen peroxides activity), Caro (carotenoids), chl *b* (chlorophyll *b*), Chl *a* (chlorophyll *a*), TC (total chlorophyll), pods/pla (pods per plants), seeds/pod (seeds per plants), SVI (seed vigor index), N (nitrogen), P (phosphorus), K (potassium), Pro (proline content in radish plants), Pb in root (lead in radish roots), Pb in shoot (lead in radish shoot), AC (accumulation coefficient), MTI (metal tolerance index), GP (germination percentage).

The cultivation of *R. sativus* on soil contaminated with Pb, supplemented with PEG, exhibited a correlation between growth and various physiological and biochemical traits. This relationship was elucidated through the utilization of loading plots derived from principal component analysis, as depicted in Fig. 5. The initial two principal components, Dim1 and Dim2, constitute the predominant proportion of all components and encompass over 79.0% of the entire dataset. Dimension 1 constitutes 57.2% of the dataset, while Dimension 2 accounts for 21.8% of the dataset. A positive correlation was observed among various variables, including potassium, magnesium, sodium, zinc, total phenol content and stomatal conductance. The proline content, metal tolerance index and accumulation coefficient exhibited a significant negative correlation. The largest impact on the development and antioxidant capacity of *R. sativus* was seen under Pb stress. Plant growth was negatively correlated with it, but antioxidant capacity was positively correlated. PEG primarily increased plant height, aboveground biomass and POD, CAT and SOD activity to boost plant growth and antioxidant capacity (Fig. 5).

## Discussion

Lead (Pb) is a common pollutant and its increased contamination has alarmed the researchers to find ways to reduce the toxicity impacts of this heavy metal on our ecosystem. Priyanka et al. (2021) revealed that Pb pollution reduces crop growth and productivity. According to Diaconu et al. (2020) plants acquire Pb through their root system, with a minor uptake occurring through the leaves. Primarily Pb is accumulated in the root system. However, a fraction of this element is also transported to the aboveground plant parts including foliage, shoot and reproductive tissues (Yadav et al., 2022; Bassegio et al., 2020). Upon entering into cellular compartments, Pb elicits inhibitory effects on the actions of various growth-related enzymes (Shu et al., 2012). Reduced enzymatic activity instigates perturbations in mineral nutrition and the equilibrium of water homeostasis. Furthermore, Pb induces modifications in hormonal synthesis exerting noticeable impacts on the structural integrity and permeability of cellular membranes (Zulfiqar, Akram & Ashraf, 2020; Usman et al., 2020). These multifaceted cellular responses highlight the harmful influence of Pb on various physiological processes, as validated by the pertinent literature.

During the present study, radish seeds were cultivated in soil contaminated with Pb to systematically evaluate the consequences of Pb toxicity on seed germination and subsequent plant growth. Our study unveiled a detrimental impact of Pb toxicity on compromised seed germination, reduced seed vigor index and diminished weights of both roots and shoots. A significant decrease in germination percentage (Table 1) of seeds growing in Pb spiked soil correlates with the findings of Nas & Ali (2018). Sengar et al. (2008) and Kadoll (2021) also described the same inhibitory effect of heavy metals on the germination percentage of *Oryza sativa, Spartina alterniflora, Z. mays* and *Hordeum vulgare.* The presence of Pb also affects the cytokinesis by disrupting microtubules leading to a decline in cell division (Nabi et al., 2019) resulting in decreased germination percentage and seedling vigor.

Pb imposes deleterious impacts on food reserves and causes a significant change in the enzymatic activity of amylases and proteases which are involved in seed germination of numerous higher plants (Osman & Fadhlallah, 2023; Singh et al., 2011; Sengar et al., 2008; Yang et al., 2020).

On the other hand, Galhaut et al. (2014) described the alleviative activity of PEG against Pb-induced toxic effects. They reported that increased germination rate and seedling growth in PEG-primed seeds correlated with our findings. In our experiment, the stress-ameliorative potential of 30% PEG was exhibited through improved chlorophyll synthesis in radish plants under Pb stress (Table 2). Laxman et al. (2020) and Pagano et al. (2023) also described the efficacy of PEG in enhancing seed germination percentage ascribed to modification of seed water relationship. Our findings in the context of improved germination are also similar to the results of Nagarajan et al. (2005) which showed an increased germination percentage of seeds in tomatoes when primed with PEG as priming with PEG enhanced tissue hydration of seeds which increased the efficacy of seeds against stress agents.

Lead accumulation causes a disturbance in cellular division and elongation resulting in reduced plant growth and biomass accumulation (Khan et al., 2020; Diaconu et al., 2020) which are allied with our current findings (Table 1). Data presented in Table 1 showed decreased root dry weight in radish plants exposed to Pb. Similar findings were reported in the case of wheat, cotton, sunflower and brassica subjected to metal stress (Ramana et al., 2021; Kanwal et al., 2020; Bassegio et al., 2020; Alaboudi, Ahmed & Brodie, 2018). Ghori et al. (2019) reported that a reduction in biomass and growth of plants is associated with a decline in photosynthetic activity resulting from retardation in the activity of photosystem II and nitrogen assimilation which is in agreement with our findings (Tables 2 and 3).

The present study elucidates the pronounced influence of Pb on gas exchange parameters, corroborating earlier studies that consistently underscore the deleterious effects of this noxious metal on the photosynthetic rate (Lamhamdi et al., 2013). This phenomenon is attributed to disruptions in the chloroplast ultrastructure, diminished chlorophyll synthesis, interference with electron transport and inhibition of crucial enzymes in the Calvin cycle. The closure of stomatal pores leads to a reduction in stomatal conductance, carbon dioxide levels and rate of photosynthesis is greatly responsible for reduced rates of transpiration has been evidenced and substitution of Mg^2+^ ions within the chlorophyll molecule induces structural damage as it is a pivotal component of chloroplast, thereby diminishing photosynthetic efficiency. Therefore, the results of our study correlating with the former investigations showed reduced stomatal conductance, chlorophyll content (Table 2) and photosynthetic efficiency (Fig. 1). Similarly, the affinity of Pb toward proteins essential for light reactions, Calvin cycle and in photosystems adversely affects the photosynthetic apparatus, consistent with prior studies demonstrating alterations in chlorophyll content in *Spinacia oleracea* (Lamhamdi et al., 2013) under Pb toxicity. Additionally, activation of chlorophyllase, excessive ROS accumulation, inhibition of 8-aminolevulinate dehydrogenase and alterations in thylakoid membrane lipid composition contribute to reduced chlorophyll levels and all these significant outcomes of former research work suggest the reason for reduced photosynthesis and chlorophyll content in our current findings. The present study showed enhanced photosynthetic activity in radish plants primed with PEG under Pb stress which aligned with the study of Salah et al. (2015) who described an increased photosynthetic pigment level in rice seeds primed with PEG.

Rizvi et al. (2020) showed a reduced absorption of essential elements that play a crucial role in the metabolism of plant biomass production in Pb-contaminated regimes that is by reduced N, P and K uptake in the seedlings grown in the Pb regime. Our data exhibited that Pb toxicity diminished the concentrations of N, P and K in various tissues of radish plants. Kanwal et al. (2020) also observed a decreased level of N in plants subjected to higher concentrations of Cd, Pb and Zn. HM’s induced stress affects N uptake, transportation and assimilation in plant tissues Laxman et al. (2020) The adverse impact of Pb on ionic homeostasis declines K uptake and translocation, resulting in poor plant growth (Naciri et al., 2021) which correlates our study in reduced uptake of minerals (Table 3).

Proline, an amino acid that accumulates in response to Pb toxicity, is essential for the induction of plant defense mechanisms (Mishra et al., 2006). The higher accumulation of proline is proposed to function as a protective mechanism, particularly for proteins involved in cellular redox maintenance and proline playing a role as a beneficial solute is suggested to be involved in the synthesis of proteins. These findings align with the outcomes of our current investigation as shown in Fig. 3.

The presence of Pb perhaps led to a significant reduction in soluble protein concentration compared to stress-free conditions. Comparable findings were reported by Xalxo & Keshavkant (2020) in their study on *Talinum triangulare*, suggesting that diminished protein content may result from suppressed synthesis and increased oxidation induced by Pb toxicity.

Our data showed that radish plants growing in Pb-spiked soils exhibited higher levels of ROS, proline, hydrogen peroxide, MDA and antioxidants which are aligned with studies of Priyanka et al. (2021) and Yan et al. (2021), illustrating that HM’s induced stress elevated lipid peroxidation, disrupted cellular metabolic homeostasis and triggered free radical production. Higher ROS generation stimulated non-enzymatic and enzymatic antioxidant mechanisms as adaptive responses to mitigate metal stress (Sharma et al., 2020). The outcome of our study aligns with the observations made by Sidhu et al. (2016), who reported an increase in the antioxidant activities of *Lepidium coronopus* when exposed to Pb. Similarly, in a study conducted on *Zygophyllum fabago*, it was observed that exposure to Pb resulted in elevated levels of catalase (Salah et al., 2015). Taken together, the results of our study showed that PEG may act as a secondary messenger to alleviate the metal-induced stress in plants. Seed priming with PEG mitigated Pb stress in radishes through decreasing Pb uptake, increasing chlorophyll content and regulating synthesis and/or activation of enzymatic and non-enzymatic antioxidants which is in resemblance with the outcomes of Syaiful et al. (2015) explained that priming with PEG increases membranes stability and effective translocation of amino acids, soluble sugars and proteins which probably enhance stress tolerance ability. Nevertheless, the stress ameliorative action of signaling molecules triggered by PEG to mitigate Pb-induced toxicity remains mysterious. Hence, further studies are required to recognize the precise mechanism of the PEG-induced extenuation of Pb toxicity through increasing the physiological, biochemical, molecular and growth tactics.

## Conclusion

In conclusion, Pb toxicity negatively affected physiochemical activity, gas exchange parameters, germination, nutrition and growth of radish. Pb-induced stress at the same time activated the tolerance mechanism of plants. However, plants remained incapable of managing Pb-triggered cell injuries as perceived through the decreased synthesis of photosynthetic pigments, root growth, shoot growth and biomass production. Pb-stressed plants exhibited elevated levels of proline biosynthesis besides enhanced activity of antioxidative enzymes. Yet, 30% PEG seed priming abridged the stress injuries through increased activity of SOD, POD and CAT enzymes besides increasing the scavenging level of H_2_O_2_ and MDA. Similarly, PEG improved photosynthetic activity by protecting the deterioration of photosynthetic pigments by adjusting water equilibrium. The modulated activity of antioxidant enzymes, enhanced biosynthesis of soluble proteins and higher production of osmoprotectants (proline) and total soluble sugar through PEG seed application maintained cellular osmotica and cellular integrity. Moreover, PEG-applied plants showed decreased Pb accretion and increased levels of mineral nutrients (N, P, K). Collectively, 30% PEG seed priming enhanced nutrition, gas attributes, photosynthetic activity, growth and biomass production of radish under Pb stress. Further detailed biosynthesis pathways and omics modulations studies related to the stress tolerance mechanism through PEG application should be explored. A minute amount of stress ameliorator is required for seed priming. Hence, this approach, exhibiting promising results in further field trials, might be extensively employed for crop production under abiotic stress conditions. Moreover, the findings of this study may aid in making breeding selections for developing abiotic stress-tolerant varieties.

## Supplemental Information

10.7717/peerj.18147/supp-1 Supplemental Information 1 The data obtained represents the mean value of three replicates RStudio software was employed to conduct principal component analysis (PCA) and determine Pearson correlation coefficients among the observed variables. SPSS Ver. 20 was used to calculate the analysis of variance. Subsequently, the Tukey Honestly Significant Difference (HSD) test was employed to assess the treatment means, enabling the ranking and comparison of significantly distinct treatments at a probability level of *P* < 0.05.

## Additional Information and Declarations

### Data Availability

The following information was supplied regarding data availability: The raw data is available in the Supplementary File.
